# HBV genotypes and response to tenofovir disoproxil fumarate in HIV/HBV-coinfected persons

**DOI:** 10.1186/s12876-015-0308-0

**Published:** 2015-07-08

**Authors:** Florian Bihl, Gladys Martinetti, Gilles Wandeler, Rainer Weber, Bruno Ledergeber, Alexandra Calmy, Manuel Battegay, Matthias Cavassini, Pietro Vernazza, Anna-Paola Caminada, Martin Rickenbach, Enos Bernasconi, Swiss HIV Cohort Study

**Affiliations:** 1 Cantonal Hepatobiliary Unit, Ente Ospedaliera Cantonale, Ospedale San Giovanni Bellinzona,Switzerland and Gastroenterology and Hepatology Service, University Hospital of Geneva, Geneva, Switzerland; 2 Institute of Microbiology, Ente Ospedaliera Cantonale, Bellinzona, Switzerland; 3 Department of Infectious Diseases, University Hospital of Bern, and Institute of Social and Preventive Medicine, University of Bern, Bern, Switzerland; 4 Division of Infectious Diseases and Hospital Epidemiology, University Hospital of Zurich, University of Zurich, Zurich, Switzerland; 5 Division of Infectious Diseases, University Hospital of Geneva, Geneva, Switzerland; 6 Division of Infectious Diseases, University Hospital of Basel, Basel, Switzerland; 7 Division of Infectious Diseases, University Hospital of Lausanne, Lausanne, Switzerland; 8 Division of Infectious Diseases, Cantonal Hospital of S. Gallen, St. Gallen, Switzerland; 9 SHCS Datacenter, Lausanne, Switzerland; 10 Division of Infectious Diseases, Regional Hospital of Lugano, Lugano, Switzerland

## Abstract

**Background:**

Hepatitis B virus (HBV) genotypes can influence treatment outcome in HBV-monoinfected and human immunodeficiency virus (HIV)/HBV-coinfected patients. Tenofovir disoproxil fumarate (TDF) plays a pivotal role in antiretroviral therapy (ART) of HIV/HBV-coinfected patients. The influence of HBV genotypes on the response to antiviral drugs, particularly TDF, is poorly understood.

**Methods:**

HIV/HBV-co-infected participants with detectable HBV DNA prior to TDF therapy were selected from the Swiss HIV Cohort Study. HBV genotypes were identified and resistance testing was performed prior to antiviral therapy, and in patients with delayed treatment response (>6 months). The efficacy of TDF to suppress HBV (HBV DNA <20 IU/mL) and the influence of HBV genotypes were determined.

**Results:**

143 HIV/HBV-coinfected participants with detectable HBV DNA were identified. The predominant HBV genotypes were A (82 patients, 57 %); and D (35 patients, 24 %); 20 patients (14 %) were infected with multiple genotypes (3 % A + D and 11 % A + G); and genotypes B, C and E were each present in two patients (1 %). TDF completely suppressed HBV DNA in 131 patients (92 %) within 6 months; and in 12 patients (8 %), HBV DNA suppression was delayed. No HBV resistance mutations to TDF were found in patients with delayed response, but all were infected with HBV genotype A (among these, 5 patients with genotype A + G), and all had previously been exposed to lamivudine.

**Conclusion:**

In HIV/HBV-coinfected patients, infection with multiple HBV genotypes was more frequent than previously reported. The large majority of patients had an undetectable HBV viral load at six months of TDF-containing ART. In patients without viral suppression, no TDF-related resistance mutations were found. The role of specific genotypes and prior lamivudine treatment in the delayed response to TDF warrant further investigation.

## Background

Hepatitis B virus (HBV) infection is one of the most frequent causes of end-stage liver disease. Due to shared modes of transmission, coinfection with human immunodeficiency virus (HIV) and HBV is frequent, and represents a global public health challenge [1]. HIV/HBV-coinfected patients, particularly those with low CD4 cell counts, are at increased risk for liver-related mortality [2–5]. Progression of HBV-related liver disease is influenced by viral factors, such as viral load, genotype, the presence of hepatitis B e antigen (HBeAg), and the appearance of escape mutations [6–11].

The natural history of HBV infection in HIV-coinfected individuals is modified by [5, 12]. nucleos(t)ide analogues (NUCs), such as lamivudine (LAM), tenofovir disoproxil fumarate (TDF), and emtricitabine (FTC), if used as components of antiretroviral therapy (ART). Moreover, recent studies showed that these HBV-specific agents not only prevent liver deterioration, but can also lead to seroconversion of HBeAg and even hepatitis B surface antigen (HBsAg) [12]. TDF, alone or combined with FTC, is a recommended component of an ART regimen for HIV/HBV-coinfected patients because of its potent antiviral activity against HIV and HBV and its excellent resistance profile [13–15]. Indeed, no convincing evidence of HBV resistance mutations against TDF has been described to date [16]. Nevertheless, TDF and FTC fail to completely suppress HBV viral load in some HIV/HBV-coinfected patients [17, 18]. A recent long-term analysis of the effects of TDF on HIV/HBV over 55 months showed a lack of virological response in 10 % of patients, who subsequently required entecavir rescue therapy [18].

HBV is classified into eight genotypes, designated A to H, based on a sequence polymorphism of more than 8 % [19]. In HBV mono-infection, viral genotypes have diverse geographical distribution, different routes of infection, and are associated with distinct disease progression and treatment responses [20–22]. HBV genotype distribution in HIV/HBV coinfection appears to be influenced by the route of infection, and to interfere with the natural history of liver disease [6, 9–11, 23]. Importantly, some HBV genotypes may hamper the efficacy of NUCs in HBV-mono-infected patients [21]. We aimed to evaluate the impact of HBV genotype on the efficacy of TDF in the treatment of HIV/HBV-coinfected patients.

## Methods

### Patient population

All patients were adults and were enrolled in the Swiss HIV Cohort Study (SHCS), which is a large prospective cohort study with continuous enrolment of HIV-infected individuals and followed in HIV outpatient clinics at 7 Swiss hospitals (Basel, Bern, Geneva, Lausanne, Lugano, St. Gallen, Zurich) [24]. The institutional review board of the Hospital where patients were recruited (Zurich, Basel, Bern, Geneva, Lausanne, Lugano and St. Gallen) approved the study and all subjects signed an informed consent before enrollment in the study. Patients were followed every six months with a study visit during which laboratory parameters were measured and plasma samples taken. All data, including laboratory results, were transmitted to a data center and plasma samples were stored at −80 °C. For this study, the following demographic and clinical characteristics were extracted: HBsAg, anti-HBc, HBeAg, anti-HBe, anti-HDV, anti-HCV and HCV RNA viremia, sex, age, date of first HIV test, route of transmission (risk group), region of origin, ethnicity, HIV viremia, ART (on treatment, off treatment, or treatment naïve), start of first HIV treatment, CD4 cell counts, alanine transaminase (ALT), aspartate transaminase (AST), international normalized ratio (INR), total bilirubin, serum albumin and creatinine.

### HBV analyses

Stored plasma samples taken before exposure to TDF were selected and analyzed for HBV DNA. In patients with detectable HBV DNA, it was amplified, HBV genotypes identified, and resistance testing performed. DNA was extracted using a commercially available extraction kit (QIAamp DNA Mini Kit). Plasma samples of patients were screened with an HBV SYBR real-time polymerase chain reaction (PCR) analyser (realtime quantitative Cobas AmpliPrep/Cobas Taqman HBV Test v1.0, Roche SA, Switzerland) with a lower limit of detection of 20 IU/ml as described previously [25]. This preliminary PCR allowed us to screen for those samples with enough DNA to be further analyzed.

For genotyping analysis, DNA was amplified by using primers specific for the preS1/preS2 region of HBV (nucleosides 3025 to 80 from the theoretical *EcoR1* site of the 3,221-nucleotide HBV sequences) as described [26]. The expected amplification product of 277 bp was used for HBV genotyping by means of a direct sequencing analysis using an automated sequencer (ABI Prism 3100, Applied Biosystems, USA). Sequence data for the amplified region of HBV-DNA were aligned with preS1/preS2 sequences from among 80 GeneBank sequences of known genotype representing eight HBV genotypes. Alignment of the sequences was performed with ClustalX software (version 1.81). Phylogenetic trees were constructed by the UPGMA method with MEGA (version 4.0) [27]. If phylogenetic analysis did not clearly determine the genotype, mostly due to insufficient quality of the generating sequences, a commercial line probe assay (InnoLiPa HBV genotype v2.0 assay, Innogenetics, Ghent, Belgium), which determines the HBV genotype using specific sequences of the S region of the HBV genome, was used to validate the genotype.

For monitoring of drug resistance amplification of DNA with a nested PCR approach was used. DNA of the HBpol region was amplified with primers described by Stuyver and coworkers [28], and sequenced as described above. Data were analyzed using HepSEQ database, a web-accessible, quality-based, molecular, clinical and epidemiological database for hepatitis B. While analyzing the nested amplification product of 341 bp long, the HepSEQ database assigns genotype and annotates known resistance mutations.

If genotype analysis using both methods did not match, generating genotype A on the preS region and genotype G on the HBpol region, involvement of mixed genotype infections was supposed. Primers described by Osiowy et al. on the preS region contain mismatching nucleotides for genotype G at the 3′ end and therefore in mixed infections, amplification of genotype G was poorer comparing to genotype A [29]. Based on HBV sequences on GeneBank, a new forward primer was designed to selectively amplify genotype G on the preS region (100 % matching) to be used in addition of preS1R as reverse primer. The sequence of this sense primer pre S1GF was 5′-AGGTAGGAGTTGGAGCCTATGG-3′. Amplifications conditions remained the same as described and both genotypes could be amplified.

Three patients showing mixed infections (genotype A and D) with preS and HBpol region were confirmed to be mixed infection with commercial Inno-Lipa HBV genotyping.

### Efficacy of TDF on HBV suppression

The efficacy of TDF to suppress HBV was defined as HBV DNA below the limit of detection (<20 IU/mL) as assessed with patients belonging to the Cohort on the routine basis, within six month from start of treatment. In patients where complete HBV suppression was not achieved with TDF, HBV subtype and treatment history were determined and repeated post-TDF plasma samples were analyzed for HBV viremia and genotypic resistance.

### Statistical methods

Fisher’s exact test and non-parametric Mann–Whitney U-test were used for comparison of categorical and continuous variables, respectively. Statistics were computed using Stata 11/SE (StataCorp LP, College Station, Texas, 77845 USA). All tests were two-tailed with significance level at 0.05.

## Results

### Analysis population and baseline characteristics

**Fig. 1:**
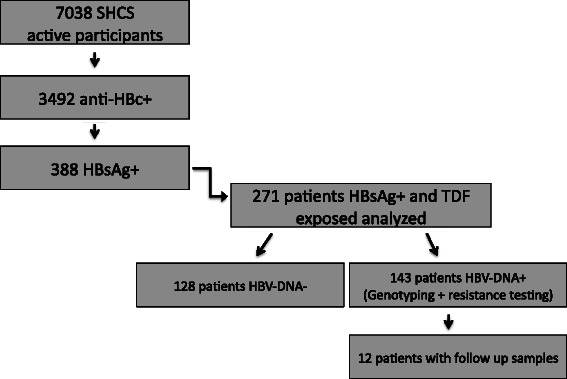
Analysis population

In 2008, a total of 15,139 participants were included in the SHCS and, of these, 7,038 were on follow-up. Of these, 3,492 (49.6 %) had anti-HBc positive status, and 388 had positive HBsAg serology (11 %). Of the HBsAg-positive participants, 271 had been exposed to TDF (Fig. 1). HBV DNA was detectable in 143 and undetectable in 128 persons before the initiation of TDF.

Baseline data for the 271 TDF-treated patients included in this analysis are summarized in Table 1. Patients with or without detectable HBV DNA were comparable; no differences were found for baseline demographic parameters (data not shown). Also, baseline clinical characteristics were similar between the two groups with the exception of median serum ALT levels of patients with detectable HBV DNA (slightly above normal at 47 IU/L (range 11–1020 IU/L, normal < 45 IU/L)), with no statistical differences observed between the genotypes.

**Table 1:** Baseline demographic and clinical characteristics of TDF-treated HIV/HBV co-infected patients

		143 patients	128 patients
		Detectable HBV DNA	Undetectable HBV DNA
Origin:	Caucasian	121 (84 %)	88 (68 %)
	African	16 (11 %)	36 (28 %)
	Asian	10 (4 %)	4 (3 %)
Age in years (median)	(range)	41 (23–73)	40 (21–75)
Sex	M:F	122 (85 %):21 (15 %)	101 (79 %):27 (21 %)
CD4 count (median)	(range)	295 (6–1143)	326 (21–1512)
HIV viremia (median) log10 cp/ml	(range)	2.84 (0–6.08)	1.74 (0–6.01)
HIV treatment	on HAART	82 (57 %)	84 (66 %)
	treatment naïve	31 (22 %)	15 (12 %)
	off HAART	30 (21 %)	29 (23 %)
Hepatitis delta infection	positive	9	4
	negative	17	8
	missing data	117	116
HCV coinfection	positive	31	37
	negative	110	87
	missing data	2	4
ALT (median) IU/ml	(range) IU/ml	47 (11–1020)	32 (7–284)
AST (median) IU/ml	(range) IU/ml	35 (19–375)	33 (14–135)
INR (median)	(range)	1 (0.9-2.6)	1 (0.9-1.2)
Total Bilirubin (median) umol/L	(range)	9 (4–243)	9 (2–30)
	(range)	39 (20–49)	39 (32–52)
	(range)	77 (37–214)	76 (41–150)

### HBV genotype distribution

HBV DNA was extracted and the HBV genotype determined in all 143 patients with detectable HBV DNA. Genotype was determined by phylogenetic analyses in 120 patients and by a commercial line probe assay in 23 patients. The predominant genotypes were A (82 patients, 57 %) and D (35 patients, 24 %). 20 patients (14 %) were infected with multiple genotypes (4 patients (3 %) A + D; 16 patients (11 %) A + G). Genotypes B, C and E were each present in two individuals (1 %) (Table 2). The genotype distribution varied among the transmission risk groups: Genotype A was most prevalent in men who have sex with men (MSM; 66 patients, 46 %), followed by the heterosexual transmission group (22 patients, 15 %), whereas genotype D was predominant in intravenous drug users (IDU; 25 patients, 17 %) (p = 0.02) (Table 2). The majority of multiple HBV genotype infections was found in MSM (13/20, 65 %) and was less common in heterosexual persons (4/20, 20 %), or in IDUs (2/20, 10 %).

**Table 2:** Distribution of HBV genotypes of TDF-treated HIV/HBV co-infected patients

Genotype	Heterosexual	IVDU	MSM	Other	Unknown
A	22	3	53	2	2
n = 82 (57 %)					
A + D	1	1	2	0	0
n = 4 (3 %)					
A + G	3	1	11	0	1
n = 16 (11 %)					
B	1	0	1	0	0
n = 2 (1 %)					
C	2	0	0	0	0
n = 2 (1 %)					
D	5	25	4	0	1
n = 35 (24 %)					
E	2	0	0	0	0
n = 2 (1 %)					
Total n = 143	36 (25 %)	30 (21 %)	71(50 %)	2 (1 %)	4 (3 %)

In one patient, a genotype D infection with a high HBV DNA level (200,000 IU/mL) was detected in 2003 before he received drugs active against HBV. Later he was treated with pegylated interferon for 6 months and, one year later, received a combination of LAM and TDF for 16 months and then stopped for a structured treatment interruption (STI) of the ART. When the STI started, HBV DNA was suppressed and telbivudine treatment was initiated; at this point the HBV genotype was A + G. Six months later, HBV DNA became slightly positive (511 IU/mL) and the detected genotype was A + G + D. No HBV resistance mutations were detected in this patient.

### HDV and HCV co-infection

HDV serology was tested in 38 patients and it was positive in 13 patients (34 %). HBV genotype was determined in 9 out of 13 HDV positive patients, 6 were genotype A and 3 genotype D. Furthermore HCV serology was known in 141 individuals; anti-HCV was identified in 31 patients (22 %) and HCV RNA was measurable in nine of these patients (median 2.6 ×10^6^ copies/mL). The majority of HIV/HCV/HBV co-infected patients (23 patients, 74 %) were HBV genotype D-infected, while a smaller group (8 patients, 26 %) was HBV genotype A-infected (4 A, 2 A + D and 2 A + G). Of the 9 viremic HCV-infected patients, 8 (89 %) had HBV genotype D and one patient had HBV genotype A + D.

### HBV genotypic resistance testing

In all 143 patients genotypic resistance testing was performed. In three patients, there was insufficient HBV DNA to perform resistance testing. Prior to TDF initiation, 89/143 patients (62 %) had wild type virus without mutations and 54/143 patients (38 %) had LAM-resistance mutations (Table 3). In follow-up samples from 12 patients who exhibited a delayed response to TDF, genotypic mutations were detected in four, although no resistance associated mutations against TDF were identified (see below).

**Table 3:** Distribution of resistance mutations prior to TDF treatment in HIV/HBV co-infected patients

	n	n HBV genotype
M204V	1	1 D
L180M + M204V	30	22 A
		5 A + G
		1 A + D
		2 D
L180M + M204I	7	3 D
		4 D
V173L + L180M + M204V	16	12 A
		1 D
		1 C
		1 A + G
		1 A + D

### Efficacy of TDF on HBV suppression

In 131 patients (92 %), TDF completely suppressed HBV DNA below the limit of detection (<20 IU/mL) within 6 months, while in 12 patients (8 %) suppression of HBV DNA was delayed (Table 4). In the 12 patients with a delayed response to TDF, HBV was detectable between 28 and 132 weeks after TDF initiation. All 12 patients were infected with HBV genotype A (5 [42 %] A + G) and previously exposed to LAM. Of the 131 patients 119 (91 %) were previously exposed to LAM, thus there is no difference in LAM exposure in the two groups (p = 0.6). In eight of the 12 patients (67 %), no genotypic resistance was detected, while in four known genotypic resistances were identified; three patients had pre-existing resistance mutations prior to TDF treatment and one exhibited a changing pattern of resistance over time (Table 4); no resistance mutations to TDF were detected.

**Table 4:** Treatment details, viremia and genotypic resistance in HIV/HBV co-infected patients with a delayed response to TDF treatment

Case	HBV genotype	HBV drug	TDF start date	weeks on TDF	HBV viremia (cp/ml)	date analyses	genotypic resistance
1	A	exposed to LAM			NA	no sample available	
		TDF	3-Jan-05		NA	no sample available	
				24	4630	21-Jun-05	L180M M204V
				101	NA	13-Dec-06	L180M M204V
				132	NA	15-Jul-07	L180M M204I
2	A + G	exposed to LAM			200000	Before TDF	No resistance
		TDF	20-May-04		200000	no sample available	
				125	99	10-Oct-06	No resistance
3	A + G	exposed to LAM			NA	no sample available	
		TDF	18-Apr-05		920	no sample available	
				116	1520	9-Jul-07	No resistance
4	A	exposed to LAM			317′000′000	Before TDF	V173L L180M M204V
		TDF	14-Mar-06		186′000′000	no sample available	
				39	273	12-Dec-06	V173L L180M M204V
		TDF + FTC	28-Jun-07	100	29	14-Feb-08	V173L L180M M204V
5	A	exposed to LAM			NA	no sample available	
		TDF	23-Aug-05		NA	no sample available	
				93	207	6-Jun-07	No resistance
6	A + G	exposed to LAM			100′000′000	Before TDF	No resistance
		TDF	18-Jan-06		1′008′000	no sample available	
7	A	exposed to LAM			793′000′000	Before TDF	V173L L180M M204I
		TDF + FTC	28-Apr-06		NA	no sample available	
				47	240	20-Mar-07	V173L L180M M204V
				73	NA	19-Sep-07	V173L L180M M204V
8	A	exposed to LAM			11′000′000	Before TDF	No resistance
		TDF + FTC	9-Nov-06	28	NA	21-May-07	V173L L180M M204V
				66	913	12-Feb-08	L180M M204V
9	A + G	exposed to LAM			NA	Before TDF	No resistance
		TDF	25-Sep-06		9′000′000	no sample available	
				43	55	23-Jul-07	No resistance
10	A	exposed to LAM			764′690′000	Before TDF	No resistance
		TDF	30-Mar-07		38′291	no sample available	
				37	252	17-Dec-07	
11	A + G	exposed to LAM			1′100′000′000	Before TDF	No resistance
		TDF + FTC	2-Feb-07		727′000	no sample available	
				36	128′000	9-Oct-07	No resistance
12	A	exposed to LAM			75	Before TDF	No resistance
		TDF	5-Feb-07		49	no sample available	
				28	NA	20-Aug-07	

One patient, who was treated for many years with LAM, harbored a L180M, M204V mutation six months after initiating TDF treatment (Table 4, Case 1). After 29 months of TDF treatment, HBV-DNA was detectable and the virus changed to a L180M, M204I genotype. Another patient was treated with LAM for 9 years (1995–2004) before it was discontinued due to HIV resistance (Table 4, case 8). In October 2006, the identified virus was wild type (no resistance) and TDF/FTC combination therapy was initiated in November 2006. Six month after initiation, the patient remained viremic and harbored a triple resistance (V173L, L180M, M204V). After a further 8 months of treatment, HBV DNA remained positive and a double mutation (L180M, M204V) was detected.

## Discussion

In the Swiss HIV Cohort study, HBV genotype A was predominant in HIV/HBVco-infected participants, followed by genotype D, which is consistent with previous reports [10, 11, 30]. There was no difference in the HBV genotype distribution between different ethnicities, but genotypes differed between HIV transmission risk groups: The majority of HBV genotype A infection was found in MSM whereas IDU was associated with genotype D, which is consistent with data from the EuroSIDA study [11]. Moreover, HBV genotype D infection was predominant in HIV/HBV/HCV triple infection (73 %), and was present in all patients with chronic HCV infection. In contrast to the EuroSIDA study, in the present study, HBV genotyping was performed by direct sequencing of the preS1 gene with subsequent phylogenetic analyses and a commercial hybridization line probe assay was used only in cases where phylogenetic analysis was inconclusive. As the results of the studies are generally in accordance it could be argued that the easier analysis with the commercial hybridization assay is as effective as the time-consuming direct sequencing approach. On the other hand, a greater number of cases of multiple HBV genotype infection were detected in our study, which could be due to the differences in sequencing approaches. In our study, multiple HBV genotype infections were identified in 20 patients (14 %), which were all in combination with genotype A (16 A + G and 4 A + D). Mixed infection with different HBV genotypes, subgenotypes and recombinations have been increasingly described especially in highly endemic Asian countries, like China [31] and Thailand [32]. In a recent study from Laos, 5.8 % of 446 HBsAg positive blood donors were infected with multiples HBV genotypes [33]. Dual infection (A + D, A + G, and D + G) has also been reported in 8 out of 241 patients (3.3 %) from Germany [34] or in a report from Canada [29].

Treatment with TDF resulted in complete suppression of HBV DNA in 92 % of patients within 6 months, and a delayed decline in HBV DNA was observed in 12 patients (8 %). No HBV resistance mutation to TDF was found to explain the delayed response, which is consistent with previous studies [16, 35]. Nevertheless, delayed viral suppression or even incomplete HBV-DNA control during prolonged antiviral treatment has been recently described with several reasons for explanation [35, 36] as poor adherence to therapy, altered HBV-specific T cell response in HIV infection [37] and the rt194T polymerase mutation [38]. However, if the viral genotype influences this setting has not yet examined. In our study, all 12 patients were HBV genotype A-infected and five (42 %) had a double HBV genotype A + G infection. In addition, all patients had been previously treated with LAM. In a recent study, a delayed response to TDF was observed in 15 % of HIV/HBV-co-infected patients who added TDF to LAM. In these patients with a delayed response to TDF no resistance to TDF was detected, which is consistent with the findings of the present study [39]. Moreover, Boyd et al., described again recently that 14 % of co-infected HIV/HBV infected patients had detectable viral load even 12 month after start of TDF without identifying specific mutations in the polymerase gene which may explain this delay [35]. Interestingly, in this study HBV genotype is available in many, but not all patients and 63 % of patient with transient viremia were genotype A and even more 77 % of patients with persistent high or low level viremia [35]. This high numbers of genotype A in “difficult to treat patients” were not further discussed but indicate towards our findings. Furthermore, a chart review of 31 HIV/HBV-coinfected patients reported a shorter time to suppression of HBV DNA in LAM-naive patients treated with TDF and FTC (n = 12) compared with patients in whom LAM had previously failed (n = 19) [40]. The median time to complete suppression of HBV was 466 days in the LAM-naive group compared with 877 days in patients who had previously received LAM (P = 0.001). After 24 months, 5/5 (100 %) LAM-naive patients had an undetectable HBV DNA level compared with 4/13 (31 %) prior-LAM patients (P = 0.015). However, the influence of HBV genotype on TDF + FTC efficacy was not reported.

Taken together, in our study we found a common virological characteristic, present in all patients that had a delayed antiviral response to TDF. The definition of HBV genotype is a sequence polymorphism difference of more than 8 % of the genome [19] and this may play a role in the immunological control [41] that is clearly needed in the antiviral treatment with TDF. Nevertheless, this hypothesis of different immune regulation needs to be confirmed in larger studies and linked to other parameters as pretreatment LAM mutations.

Our study has limitations due to the retrospective nature of the analysis. Notably, it was not possible to compare the status of liver disease (histology or transient elastography) together with the genotype distribution and correlate this with liver disease progression, as these data were not available in the database. However, the high number of coinfected patients and the accuracy of genotype analyses added strength to the study.

## Conclusion

In conclusion, these data confirm that the most frequent HBV genotype in HIV/HBV-coinfected patients is genotype A, followed by genotype D, and that the genotype distribution is associated with the risk for HIV acquisition. Our results also suggest that a higher proportion of multiple HBV genotype infections may be present in HIV/HBV-coinfected patients than previously reported. In addition, our results demonstrated complete suppression of HBV DNA in 92 % of TDF-treated patients within 6 months, yet a minority of patients (8 %) exhibited a delayed virological response to TDF but all without HBV resistance mutations. Know parameters as prior LAM treatment with selected mutations or poor drug adherence, may influence TDF response even if HBV genotypes A infection as found in this cohort could explain this delay and may have clinical impact but warrants further investigation.

## Abbreviations

HBV: Hepatitis B virus
HIV: Human Immunodeficiency virus
TDF: Tenofovir disoproxil fumarate
ETV: Emtricitabine
ART: Antiretroviral therapy
